# Characterization of RNA from Exosomes and Other Extracellular Vesicles Isolated by a Novel Spin Column-Based Method

**DOI:** 10.1371/journal.pone.0136133

**Published:** 2015-08-28

**Authors:** Daniel Enderle, Alexandra Spiel, Christine M. Coticchia, Emily Berghoff, Romy Mueller, Martin Schlumpberger, Markus Sprenger-Haussels, Jonathan M. Shaffer, Eric Lader, Johan Skog, Mikkel Noerholm

**Affiliations:** 1 Exosome Diagnostics GmbH, Am Klopferspitz 19a, 82152 Martinsried, Germany; 2 Exosome Diagnostics Inc., 840 Memorial Drive, Suite 3, Cambridge MA 02139, United States of America; 3 QIAGEN GmbH, QIAGEN Strasse 1, 40274 Hilden, Germany; 4 QIAGEN, 6951 Executive Way, Frederick, MD 21703, United States of America; Colorado State University, UNITED STATES

## Abstract

Exosomes and other extracellular vesicles (commonly referred to as EVs) have generated a lot of attention for their potential applications in both diagnostics and therapeutics. The contents of these vesicles are the subject of intense research, and the relatively recent discovery of RNA inside EVs has raised interest in the biological function of these RNAs as well as their potential as biomarkers for cancer and other diseases. Traditional ultracentrifugation-based protocols to isolate EVs are labor-intensive and subject to significant variability. Various attempts to develop methods with robust, reproducible performance have not yet been completely successful. Here, we report the development and characterization of a spin column-based method for the isolation of total RNA from EVs in serum and plasma. This method isolates highly pure RNA of equal or higher quantity compared to ultracentrifugation, with high specificity for vesicular over non-vesicular RNA. The spin columns have a capacity to handle up to 4 mL sample volume, enabling detection of low-abundance transcripts in serum and plasma. We conclude that the method is an improvement over traditional methods in providing a faster, more standardized way to achieve reliable high quality RNA preparations from EVs in biofluids such as serum and plasma. The first kit utilizing this new method has recently been made available by Qiagen as “*exoRNeasy Serum/Plasma Maxi Kit*”.

## Introduction

### Extracellular vesicles and their importance for molecular pathology

Exosomes, microvesicles, and other extracellular vesicles have been understood to exist for more than 30 years ([1] and references within), but their potential for biomarker discovery and diagnostic development has been vastly underappreciated until recently [2–4]. Since the nomenclature of these vesicles is still being discussed [5], this article will collectively refer to exosomes and other extracellular vesicles as EVs. The majority of EVs are roughly 50–200 nm in diameter and known to form by two separate mechanisms. One pathway is through inward budding of endosomal membranes, which give rise to intracellular multivesicular bodies that later fuse with the plasma membrane, releasing the EVs to the exterior of the cell [1,6]. Alternatively, vesicles can be shed directly from the cell by outward budding of the plasma membrane [7,8]. In both cases, all EVs have a lipid bilayer surrounding the internal components, protecting them from enzymatic degradation and creating a naturally stable environment.

Release of EVs from cells is an active process, and has been shown to provide a mechanism for cell-to-cell communication [9]. Tumor exosomes have been shown to mediate critical steps in the disease process, such as stimulation of angiogenesis [10] blunting the immune response [11,12] or even involvement in pre-metastatic niche formation [13]. Exosomes have also been shown to have important roles in normal physiology and pregnancy, where placental exosomes help to form an immunosuppressive barrier for the mother’s immune system [14,15]. As EVs are released during both normal and pathological conditions, their protein and lipid components have been extensively described [16,17]. However their nucleic acid components have only recently been the subject of study. EVs contain RNA [2,18] as well as DNA [19,20]. The RNA component of EVs is of particular diagnostic interest, as naked RNAs are not stable in blood outside of the vesicles, due to exposure to RNases [21]. Not all stable RNA in biofluids is contained within vesicles, however. There are miRNAs protected by protein complexes containing Ago2 [22–24] and some miRNAs have also been associated with HDL and LDL particles [25]. The RNA content of EVs has been described using RNA-seq, hybridization arrays and other screening methods, revealing the full spectrum of previously known transcript types. This includes miRNAs and other species of small non-coding RNAs (ncRNAs) like piRNAs, but also mRNAs, tRNAs, lncRNAs and rRNAs [26–28]. Moreover, there is intriguing evidence for vesicle-specific modification, enrichment, and isoforms of the enclosed RNA [2,26,29].

Reproducible RNA extraction from biofluids allows analysis of gene expression changes during disease. While mutation detection can be done at both the DNA and RNA level, many biomarkers are uniquely analyzed on the RNA. For example, transcriptional changes are measured at the RNA level, such as spatial or temporal deregulation of mRNA, miRNA, lncRNA and other non-coding RNAs. There are also disease specific splice variants (*ARV-7* in prostate cancer [30], *EGFRvIII* in glioblastoma [31], *BRAF* variants in melanoma [32]) and larger deletions/fusions in intronic DNA regions that are more easily detected on the RNA level where the fusion site is defined, i.e. *ALK* fusions in lung cancer [33]. Thus, RNA analysis an attractive strategy for diagnostic development [34]. Here, the protective bilayer of EVs makes it possible to use both fresh and archived samples for RNA analysis; samples frozen for more than twelve years can still yield high-quality RNA ([2], S2 Fig).

Development of biofluid-based biomarkers would limit the need for tissue biopsies and, with a simple non-invasive collection, enables longitudinal monitoring of health and disease. In Oncology, biofluids might also provide a more accurate representation of a tumor’s genetic makeup than a spatially restricted fine needle biopsy, including the heterogeneity of the primary tumor and of metastatic lesions [35,36]. Currently, there are methods to examine DNA-based biomarkers in biofluids, either by isolating circulating tumor cells (CTCs) or circulating cell-free DNA (cfDNA) [37]. Analyzing the genome of single CTCs allows to call somatic mutations [38], CTC abundance in blood is used for an FDA-cleared assay for cancer progression (CELLSEARCH) and cfDNA already allows to detect mutations in blood from a variety of cancers [39–41]. The development of RNA-based biofluid diagnostics utilizing EVs, however, is impeded by the lack of a standardized RNA isolation method with robust, reproducible performance.

### Current standard of RNA extraction from EVs

Historically, the ‘gold standard’ for isolating EVs, and the enclosed RNA within, with good yield and quality has been differential ultracentrifugation. This is a time-consuming process, requiring a well-optimized protocol and significant capital investment. Unfortunately there is no standardization of these protocols, and laboratories are using different pre-processing protocols (*i*.*e*. generation of platelet poor plasma or removal of the “cell debris”) as well as different protocols for ultracentrifugation itself. Even where attempts are made to keep the spin times and centrifugal force constant, the pelleting efficiencies vary since different labs often use different rotors (with varying k-factors) and different sample dilutions steps [42]. These variables make comparison of results between studies virtually impossible and there is a need for more easily standardized methods. Due to such protocol discrepancies, the actual RNA content of EVs is still actively discussed. Development of a robust method that yields RNA in quality and quantity similar to ultracentrifugation has come to be recognized as an ideal solution, and many laboratories have endeavored to develop such a method, using a variety of approaches [43]. Methods using size-based filters (*e*.*g*. ExoMir, Biooscientific), antibody-based capture (*e*.*g*. Immunobeads, HansaBioMed), and polymer-based precipitation reagents [44] (*e*.*g*. Life Technologies, System Biosciences Inc.) are all available for researchers, providing varying degrees of utility. For example, kits developed with size-based filters lack specificity for the EV fraction; any particle matching the size of the filter will be retained by it, including cellular debris, protein complexes, and even platelets and lymphocytes, depending on the filter size. Polymer-based precipitation and direct lysis using chaotropic salts also suffer from a lack of specificity for vesicles. Here, undesired protein-bound extracellular RNA is prone to co-precipitate and be co-isolated [45]. Antibody-based purification of EVs has the potential to be very specific, but relies heavily on prior knowledge of EV protein content, conflicting with the still mostly unknown biology of EVs.

Here we characterize the performance of a recently developed spin column-based method to isolate EVs and extract the RNA contents from plasma and serum in an easy and reproducible workflow. The method was extensively compared to an optimized ultracentrifugation procedure as the current gold standard for EV isolation. RNA integrity and size distribution were assessed by Bioanalyzer electrophoresis; purity, yield, and composition by RT-qPCR. The method also allows for intact vesicles to be eluted from the column material, which we used for examination of the isolated EVs by electron microscopy. This new procedure captures nearly 100% of mRNA from plasma samples and is equal to or better than ultracentrifugation in mRNA yield. In addition, the method allows a fraction of miRNAs, previously shown to be “free circulating” (cell-free, non-vesicular) and associated with Ago2-protein complexes [22], to pass through the filter and remain in the flow-through. The procedure is easily adapted to clinical laboratory workflows and facilitates the recovery of total RNA content of the EVs.

## Materials and Methods

### Standard protocol for isolating EVs using membrane affinity columns

**Fig 1:**
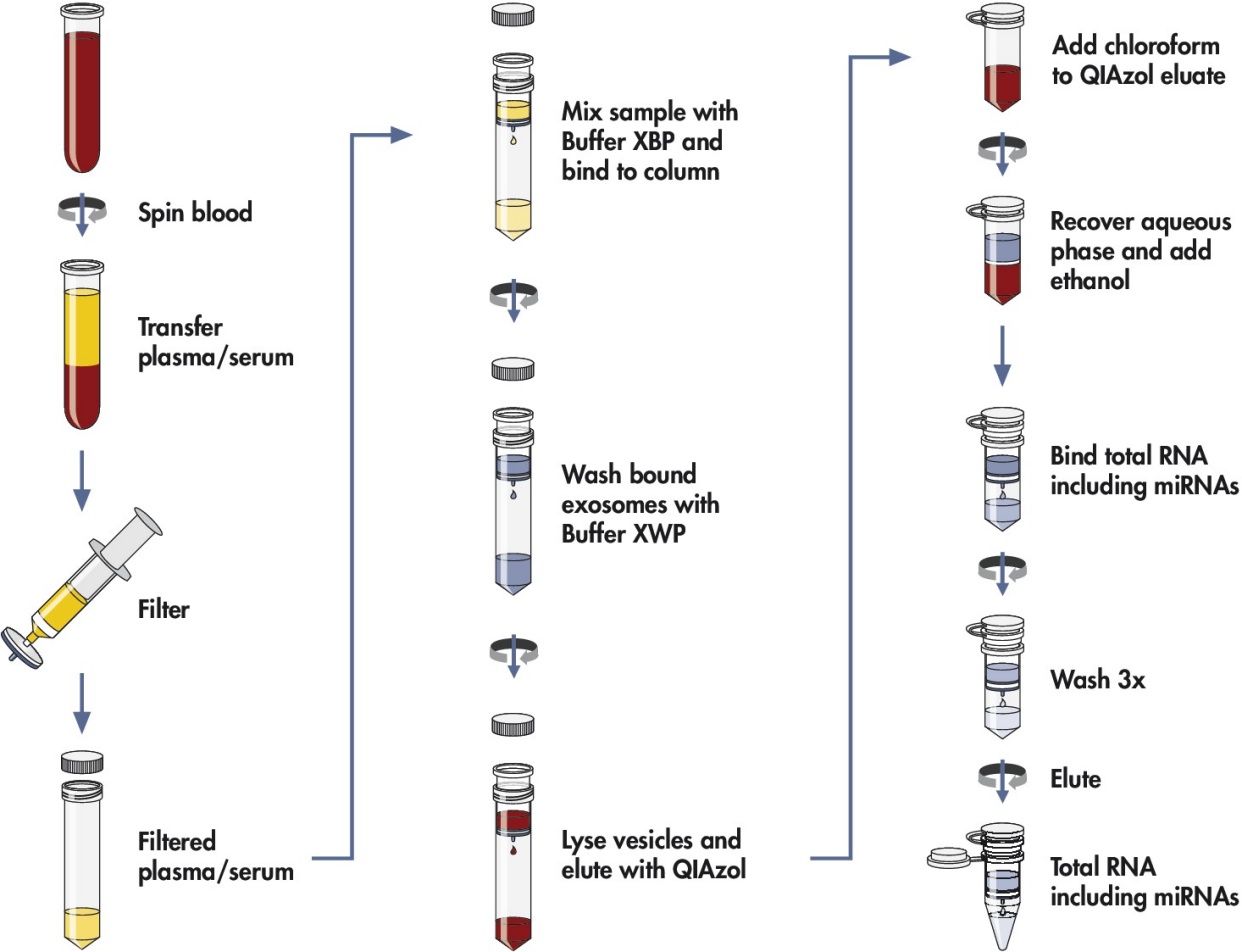
Workflow for isolating RNA from extracellular vesicles using membrane affinity columns. EV RNA is isolated from whole blood by separating the plasma or serum, pre-filtering the sample to exclude cell-contamination, and loading on the membrane affinity column followed by a brief wash. The bound vesicles are lysed and eluted with QIAzol; the RNA extracted by addition of chloroform, precipitated by ethanol and further purified using an RNeasy column.

The newly developed method purifies EVs from biofluids by a spin column-based procedure using affinity membrane binding of all extracellular vesicles, including exosomes, as illustrated in Fig 1. For compatible plasma and serum tubes, please refer to the corresponding methods section below.

The method is being distributed by QIAGEN (QIAGEN GmbH, Hilden, Germany) as *exoRNeasy Serum/Plasma Maxi Kit*, and the standard protocol is described in the *exoRNeasy Serum/Plasma Handbook*. Briefly, prefiltered plasma was mixed 1:1 with 2x binding buffer (XBP) and added to the exoEasy membrane affinity column to bind the EVs to the membrane. After centrifugation, the flow-through was discarded and wash buffer (XWP) was added to the column to wash off non-specifically retained material. After another centrifugation and discarding of the flow-through, the vesicles were lysed by adding QIAzol to the spin column, and the lysate was collected by centrifugation. The *miRNeasy Serum/Plasma Spike-In Contro*l (QIAGEN, Hilden) was added. Following addition of chloroform, thorough mixing and centrifugation to separate organic and aqueous phases, the aqueous phase was recovered and mixed with ethanol. The sample-ethanol mixture was added to an RNeasy MinElute spin column and centrifuged. The column was washed once with buffer RWT, and then twice with buffer RPE followed by elution of RNA in water. This procedure allows concentrating the extracellular RNA from 4 mL plasma or serum into a final volume of 14 μL of water.

### Serum and plasma sources

All serum or plasma was pre-filtered through a 0.8 μm syringe filter prior to EV processing. We used both single-donor / single blood-draw normal healthy control samples, samples from patients with colorectal, melanoma, lung, and breast cancer, as well as pools of plasma or serum from multiple blood donors. Although there are differences between donors and sample types, we never observed any difference in performance of the affinity membrane method between these samples. In a dedicated effort to ensure the compatibility of the method with various types of common blood tubes, we tested several serum collection tubes (e.g. BD#368815; BD#367953), plasma collection tubes containing EDTA (e.g. BD#367941, BD#362799) and citrate (e.g. BD#366575) in direct comparison from the same single-draw from several donors (data not shown). The only tube type that showed reduced performance was the heparin tube, which is known to be problematic in molecular biology applications due to the inhibitory effects of heparin on enzyme activity in downstream processes. The data presented in the following is typical data from either of the normal control sources mentioned above.

### Ethics statement

All plasma samples used in the presented work were collected with full written informed consent. Healthy control samples were received from Bavarian Red Cross under approval of the ethics review board “Ethikkommission der Bayerischen Landesaärztekammer” and melanoma samples were collected at the LMU Munich under approval of the ethics review board “Ethikkommission der Medizinischen Fakultät der Ludwig-Maximillians-Universität München” from Feb 9, 2011 (Proj No. 437–10).

### Elution of EVs from the membrane affinity column

**Fig 2:**
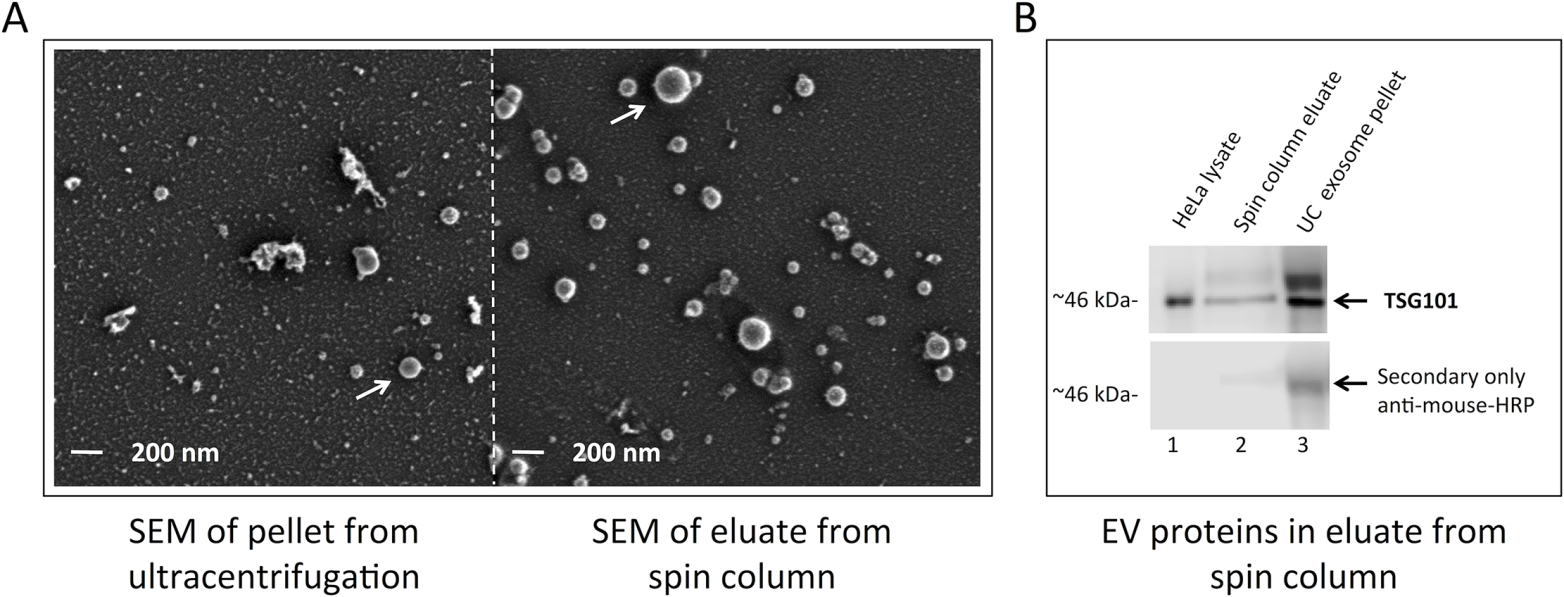
Scanning electron microscopy and western blot analysis of intact vesicles isolated by membrane affinity capture and ultracentrifugation. (**A**) Scanning electron microscopy (SEM; 20.000 x magnification) of a solubilized pellet from ultracentrifugation of pre-filtered (0.8 μm) plasma compared to a non-lysed eluate from the membrane affinity spin column. Both preparations contain vesicle-shaped structures with an expected size range from 50–200 nm (white arrows; scale bar 200 nm) indicating that intact vesicles are eluted from the spin column membrane. (**B**) Exosomes were isolated from four milliliters of normal human plasma using either the membrane affinity column (lane 2) or ultracentrifugation (UC) method (lane 3). Exosomes were concentrated, washed, then lysed, and exosome protein lysates were processed as described in Materials and Methods. The signal for TSG101 runs close to the predicted molecular weight of 43 kDa, the specificity of the TSG101 antibody was confirmed by positive control HeLa cell lysates and further verified by the absence of the 46 kDa band when probed with secondary antibody only. The blot shown is a representative of at least three separate experiments, indicating that the exosome-enriched protein TSG101 is present in vesicles eluted from the membrane affinity column.

EVs from 4 mL of prefiltered plasma were isolated using a procedure modified from the exoRNeasy protocol described in the *exoRNeasy Serum/Plasma Handbook*. Before binding of vesicles to the membrane affinity column, the column was briefly washed using 5 mL wash buffer (XWP) and a 5 min spin at 500 x g in a desktop centrifuge. After binding, the column was washed with 10 mL XWP and another 5 min spin at 500 x g. Subsequently, 140 μL of 2x elution buffer was applied to the spin column membrane, incubated for 5 min, and centrifuged 5 min at 500 x g to collect the eluted EVs. For protein analysis, exosomes were eluted in 500 μl of 1 x elution buffer. Eluates containing intact vesicles were concentrated using either a 100,000 molecular weight cut-off concentrator (Sartorius, Vivaspin) or ultracentrifugation at 120,000 x G for 120 minutes (*i*.*e*. Fig 2).

### Immunoblotting

Exosome protein lysates were resolved on Tris-Glycine SDS-PAGE and transferred to polyvinylidene difluoride (Immobilon-P; Millipore) membranes. Membranes were incubated in primary antibody (Abcam anti-TSG101 clone 4A10, diluted 1:100 per manufacturer instructions) overnight at 4°C, washed three times with 0.1% TBST, incubated with secondary antibody (HRP-conjugated goat anti-mouse, Thermo Scientific, Pierce, 1:10,000) for 1 h at room temperature, washed three times and detected with enhanced chemiluminescence (PerkinElmer LLC or Life Technologies) on LI-COR C-DiGit Chemiluminescent Blot Scanner using Image Studio Digits software Ver 3.1

### Nanoparticle tracking analysis (NTA)

For each NTA analysis, 4 mL of pre-filtered plasma was subjected either to ultracentrifugation or vesicles were isolated and eluted using membrane affinity columns. Serial dilutions of each specimen were prepared in Protein LoBind tubes (Eppendorf) to prevent exosome adsorption onto the walls of the tube. Set up of NanoSight analysis is well described in a previous publication [46]. Briefly, the diluted samples were loaded into the assembled sample chamber of a NanoSight LM10, microparticles were brought into focus using the thumbprint region as a reference; 60-s video images were acquired and analyzed by NanoSight NTA 2.3 software. The values obtained for plasma and the column flow-through represent the mean and standard deviation of two replicate isolations, the values for the column eluate and the ultracentrifugation pellet represent four replicate isolations, and the values for the UC supernatant were measured with a single isolation replicate.

### RNase digestion of column-bound material

EVs were bound to a membrane affinity column using 4 mL of pre-filtered plasma and a 5 min spin at 500 x g, washed thoroughly using 18 mL of wash buffer (XWP), and cleared of excess volume by a 15 sec spin at 500 x g. Subsequently, 500 μL of a digestion mix containing either 6.25 μg/mL RNase A and/or 0.3% Triton X-100 in wash buffer (XWP) were added directly to the membrane, followed by a quick spin at 100 x g and re-application of the flow-through to soak the column material. After 30 min of incubation at room temperature with occasional swirls, the digestion mix was removed by washing the column with 18 mL of XWP. The RNA from the bound vesicles was isolated by adding 700 μL QIAzol to the column, and following the procedure detailed in the *exoRNeasy Serum/Plasma Handbook from step 7* onwards.

### EV isolation by ultracentrifugation

2–4 mL of prefiltered plasma was diluted up to 5 mL with 1x PBS in 13x41 mm polyallomer thin-wall tubes (part no. 326819, Beckman) for ultracentrifugation rotors. EVs and other small particles were pelleted in an Optima Max-XP bench top ultracentrifuge using a MLS-50 swing-out rotor (part no. 367280, Beckman) at 120,000 x g for 80 min. The supernatant was carefully aspirated by pipetting and discarded, leaving the intact pellet at the bottom of the tube. Subsequently, the pellet was either resuspended in 1x elution buffer for cryofixation and scanning electron microscopy (SEM), lysed in lysis buffer (20 mM Tris-HCL, pH 7.4, 1% Triton-X-100, 5 mM EDTA, 150 mM NaCl, 2.5 mM sodium pyrophosphate, 50 mM sodium fluoride, 1 mM sodium orthovanadate, 1 mM β-glycerophosphate, 1 mM phenylmethylsulfonyl fluoride, and 2 μg/ml each of pepstatin, leupeptin, and aprotinin) for Western Blot analysis, or incubated for 20 min in a solution containing 8 μL of RNasin Plus (40 U/μL, Promega) and 42 μL 1x PBS, when preparing the pellet for RNA isolation. The RNA was isolated by adding 700 μL QIAzol to the pellet, quickly resuspending it by pipetting, and following the procedure detailed in the *exoRNeasy Serum/Plasma Handbook* from step 7 onwards.

### Direct lysis of a plasma

RNA was isolated using the *miRNeasy Serum/Plasma Kit*, according to the manufacturer’s instructions, but with the following modifications: Samples diluted to volumes higher than 200 μL (flow-through of membrane affinity columns) were split to be processed in two separate lysis reaction following steps 1–8 of the *miRNeasy Serum/Plasma Kit* Handbook, and combining the sample by loading it on the same RNeasy column as described at step 9 of the procedure.

### Reverse transcription followed by quantitative PCR (RT-qPCR)

The complete total RNA from each RNA extraction, including extracts from the membrane affinity spin column, plasma lysis and ultracentrifugation pellets, was eluted into 16 μL of nuclease-free water. Subsequently, the RNA/cDNA input is normalized to volume of sample input, not to fluorometry or Bioanalyzer measurement. To quantify specific mRNA transcripts, 12 μL of a 16 μL RNA eluate were subjected to reverse transcription (RT) with random hexamers using the Superscript VILO cDNA Synthesis Kit according to the manufacturer’s recommendations (LifeTechnologies; part no. 11754050). For quantitative PCR, 10% of each cDNA reaction was analyzed using standard TaqMan chemistry and cycler conditions. TaqMan Gene Expression Master Mix (LifeTechnologies, part no. 4369016) was used together with the following mRNA assays: GAPDH, ID 4326317E; KRAS, ID HS00270666_m1; HPRT, ID Hs99999909_m1; RPLP0, ID 4326314E, BRAF, FWD AAAAATAGGTGATTTTGGTCTAGCTACAGT, REV TGGATCCAGACAACTGTTCAA, PROBE YAK-GATGGAGTGGGTCCCATCAG-BHQ. To analyze the quantity of specific miRNAs, 4 μL of a 16 μL eluate were subjected to specific reverse transcription using the TaqMan MicroRNA Reverse Transcription Kit (Life Technologies (part no. 14366596) according to the manufacturer’s recommendations. For quantitative PCR, 10% of each cDNA reaction was analyzed using standard TaqMan chemistry and cycler conditions. TaqMan Universal Master Mix II, no UNG (Life Technologies, part no. 4427788) was used together with the following miRNA assays: hsa-let-7a-5p, ID 000377; hsa-miR-16-5p, ID 000391; hsa-miR-92a-3p, ID 000431; hsa-miR-142-3p, ID 000464. The PCRs were performed on an ABI 7500 fast cycler with a 96 well block and the following two-step cycler profile: 50°C for 2:00 min, 95°C for 10:00 min, 40 x (95°C for 0:15 min, 60°C for 1:00 min). Raw C_T_ values are reported with a manually set threshold at 0.1 fluorescence units and automated baseline settings. Mean and standard deviation (SD) of raw C_T_ values were determined using MS Excel AVERAGE and STDEV functions, median and interquartile range were visualized by boxplots using the website http://boxplot.tyerslab.com. When performing a relative quantification, the difference of sample and control is first calculated as a delta C_T_ of the raw values (C_T_ control–C_T_ sample). Assuming a perfect efficiency of amplification, a PCR signal will double with every cycle and a delta C_T_ of 1 will correspond to a difference of 50% in PCR signal between control and sample. Based on this, the % of PCR signal can be calculated as (2^(C_T_ control–C_T_ sample))*100.

### Electrophoretic analysis of RNA

To analyze the approximate yield and size distribution of the RNA eluate, 1 μl of the eluate was subjected to the Bioanalyzer RNA 6000 Pico assay according to the manufacturer’s instructions. The aligned fluorescence trace data was exported from the instrument’s software into a CSV file and plotted using Microsoft Excel.

### Cryofixation followed by scanning electron microscopy (SEM)

For cryofixation of material eluted from the membrane affinity column, the undiluted sample was applied to a glass microscopy slide with a cover slip and snap frozen in liquid nitrogen. After removal of the cover slip the frozen specimen was fixed with 2.5% glutaraldehyde in elution buffer, washed once with elution buffer, water and dehydrated with acetone. The acetone was subsequently removed using critical point drying with liquid carbon dioxide. Samples where mounted on aluminum stubs with tempfix mounting adhesive and contacting using colloidal silver. After sputter-coating with 3–5 nm platinum the samples were examined with a Zeiss Auriga Workstation.

### RT^2^ Profiler PCR Array

The Human Cancer Pathway Finder RT^2^ Profiler PCR Array was performed following the protocol detailed in the *RT* ^*2*^ *Profiler PCR Array Handbook*. Briefly, RNA was collected from samples using membrane affinity columns. From a total eluate volume of 14 μL, 3 μL were subjected to genomic DNA elimination, cDNA synthesis and preamplification using the RT2 PCR System (PreAMP). cDNA was mixed with RT^2^ SYBR Green Mastermix and RNase-free water to make the PCR components mix, which was then distributed in 25 μl aliquots across the RT^2^ Profiler PCR Array. The plate was sealed and centrifuged to remove bubbles, then run in a thermocycler at 95°C for 10:00 min; 40 x (95°C for 0:15 min, 60°C for 1:00 min).

## Results

### The membrane affinity column captures intact vesicles

To visually inspect the vesicle population captured by the membrane affinity column, vesicles were eluted and scanning electron microscopy (SEM) was performed to micrograph the EVs, in direct comparison to a solubilized pellet from an EV isolation of the same plasma volume by ultracentrifugation. Using both methods, vesicle structures in the expected size range of 50–200 nm were clearly visible, as seen on the micrographs in Fig 2A. Interestingly, the eluate from the spin column appeared to have less granular background staining than the ultracentrifugation protocol, presumably because ultracentrifugation pellets both EVs as well as other particles whereas the membrane affinity preparation is more selective for EVs.

The isolated vesicles were also used in a western blot analysis to verify the presence of known vesicle-enriched proteins, like TSG101 [47]. Indeed, EV isolation by ultracentrifugation and spin column purification both isolated comparable amounts of TSG101 from 4 mL of plasma (Fig 2B).

### The membrane affinity column captures a vesicle population of expected size and number

**Fig 3:**
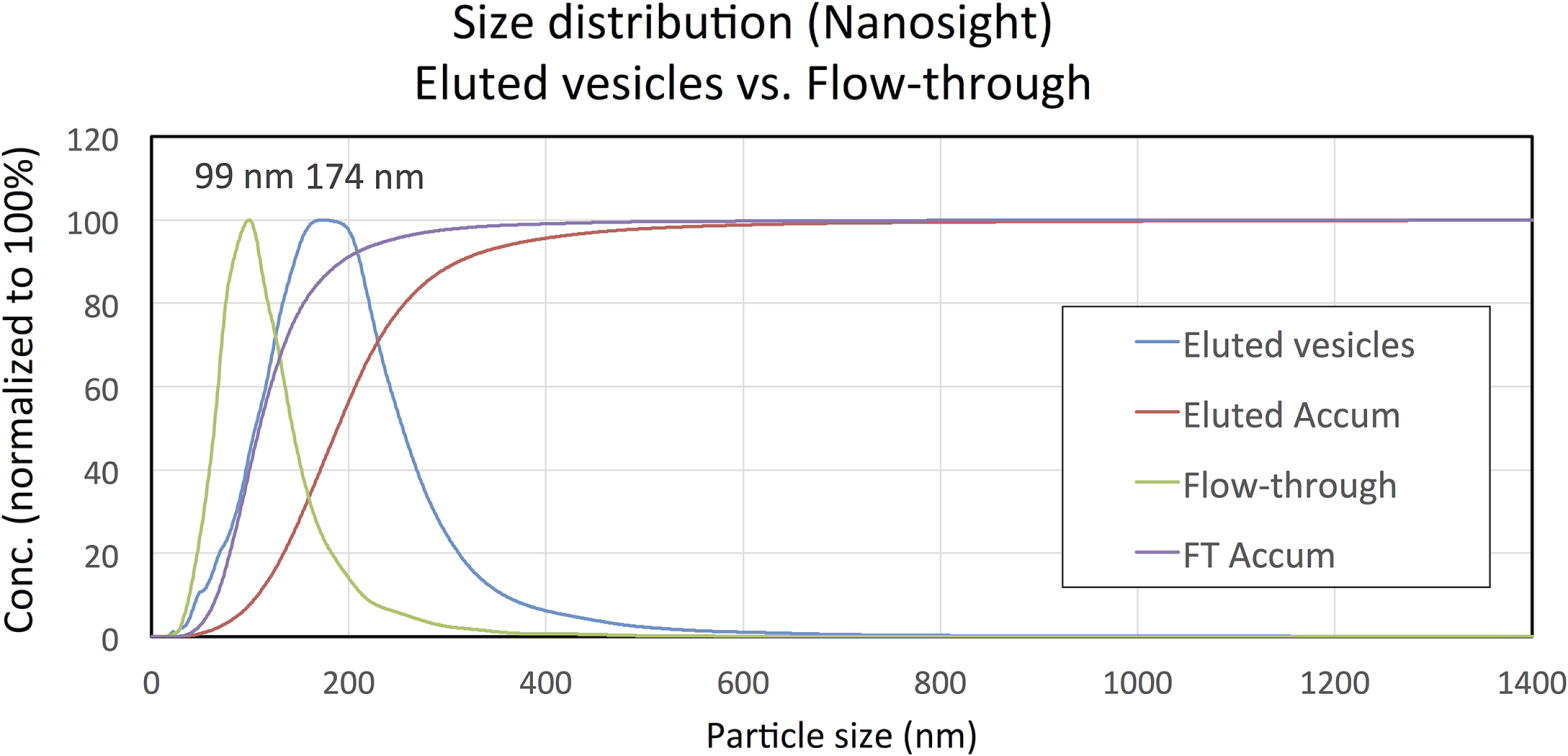
NanoSight example data of vesicles eluted from the membrane affinity column and the corresponding flow-through. Vesicles from 4 mL plasma were bound to a membrane affinity column, eluted from the column, and diluted into the working concentration of the NanoSight instrument. The flow-through from the membrane affinity column was kept and processed side by side with the eluate to compare both samples. Shown is the frequency and accumulated fractions of particle size (nm) in both samples types, differing in their average size (peak top noted in picture). While the majority of small protein complexes remains in the flow-through, leading to a small peak size of 99 nm, the eluate from the column contains vesicles, resulting in a larger peak size of 174 nm. The complete data for particle sizes and relative abundance of particles of different fractions from ultracentrifugation and membrane affinity elution is listed in Table 1 as mean and standard deviation (SD).

We further sought to qualify and compare the vesicle populations using the NanoSight instrument, which is commonly used to determine the size distribution of EV preparations. We analyzed whole plasma and compared it to the eluate from the membrane affinity column, and the corresponding flow-through from the column, as well as to a resuspended pellet from EV isolation by ultracentrifugation and the corresponding supernatant. Examples of NanoSight data are shown in Fig 3 and the values obtained from all isolations are summarized in Table 1. Although the instrument is not perfectly quantitative, the data in Table 1 clearly show that both ultracentrifugation and the membrane capture method only isolate approximately 1% of the particles present in whole plasma, whereas the majority of particles (here measured to approx. 60%) are still present in the flow-through or supernatant (the deviation from adding up to 100% is thought to be within the measurement error). Here, the majority of particles in plasma are thought to be lipoprotein particles and other large protein complexes, and both membrane capture and ultracentrifugation successfully purify the EV fraction while leaving protein particles in the column flow-through or centrifugation supernatant, respectively (Fig 3). An important limitation of the NanoSight method for quantifying EVs is the inability to distinguish vesicles from particles of similar size. However, the peak size of the particles (vesicles) from both ultracentrifugation and membrane elution was approximately the same in both vesicle preparations (173 nm and 160 nm, respectively) and clearly different from that in the flow-though (110 nm), UC supernatant (97 nm), and whole plasma (99 nm). Again, the smaller average particle size in whole plasma, flow-through, and supernatant is thought to be due to the presence of lipoprotein particles and other large protein complexes.

**Table 1:** NanoSight data comparing particles in whole plasma with fractions from membrane affinity columns and ultracentrifugation (mean ± SD). The values obtained for plasma and the column flow-through represent two replicate isolations, the values for the column eluate and the ultracentrifugation pellet represent four replicate isolations, and the values for the UC supernatant were measured with a single isolation replicate.

Protocol	Plasma (whole)	spin column eluted	spin column flow-through	UC pellet	UC supernatant
**Mean particle size (nm)**	136±6	212±12	139±13	207±16	130±NA
**Mode particle size / peak top (nm)**	99±4	160±15	110±11	173±29	97±NA
**Sample concentration (particles/mL plasma)**	4.1E+11 ±2.0E+10	5.0E+09 ±1.1E+09	2.6E+11 ±5.2E+10	3.1E+09 ±6.0E+08	2.5E+11 ±NA
**Recovery (in % of whole plasma)**	100±5%	1±0%	63±13%	1±0%	60±NA

### Isolated EVs are intact and protected from RNase activity

**Fig 4:**
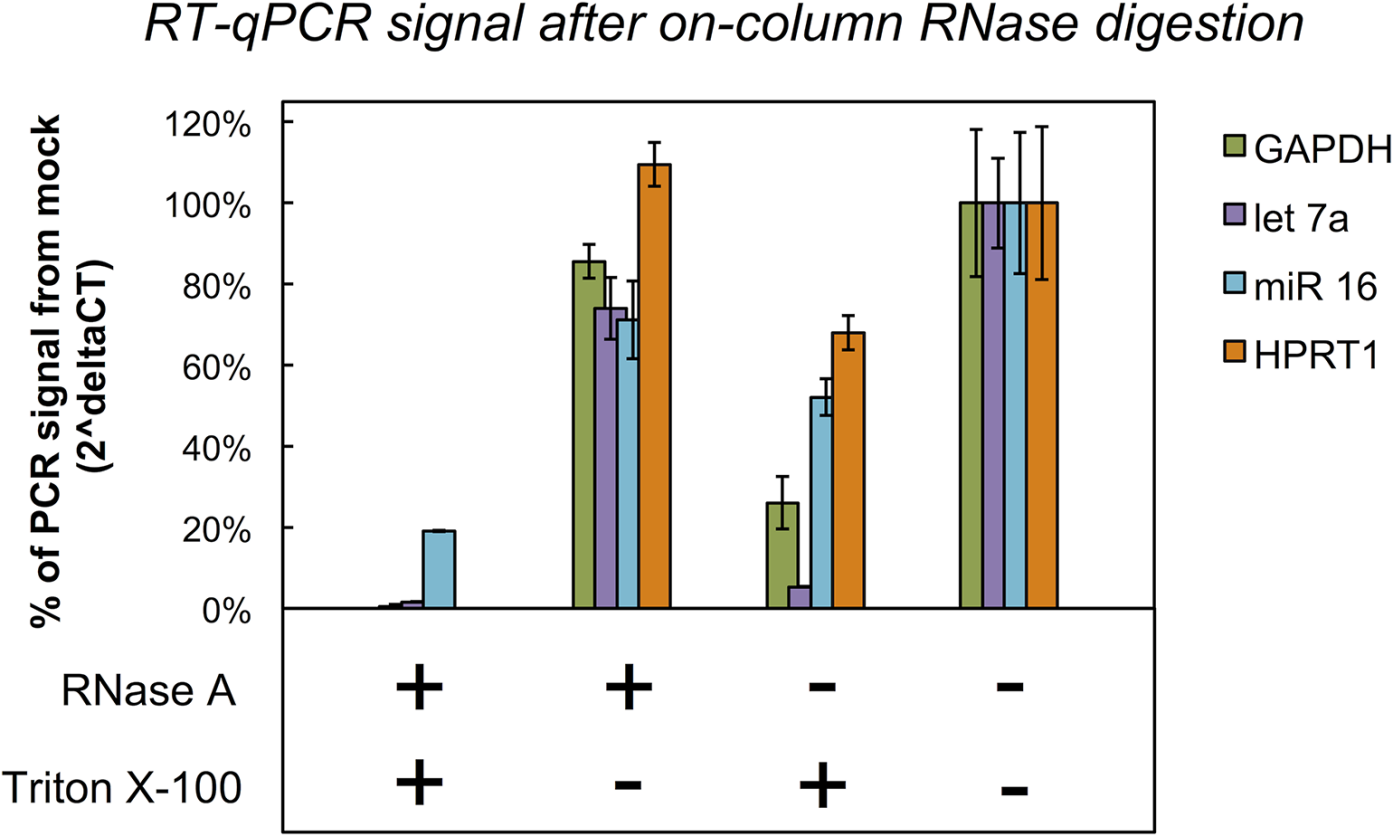
Relative quantification of column-bound RNA after treatment with RNase and/or detergent. Vesicles from 4 mL of pre-filtered plasma were bound to a membrane affinity column and washed. The column was treated for 30 minutes with either RNase A, the detergent Triton X-100, both, or reaction buffer only (mock-treatment). Subsequently, the RNA was isolated and analyzed using RT-qPCR against two mRNAs (GAPDH, HPRT1) and two miRNAs (miR-16, let-7a). The bar plots represent the relative amount of nucleic acids in the sample, compared to mock treatment alone, with columns as mean and whiskers as SD of two replicate isolations each. Assuming a perfect amplification efficiency, the % of PCR signal from mock is calculated as (2^(C_T_ control–C_T_ sample))*100 (see methods). Only when a detergent is used to destabilize the lipid-bilayers, the RNase is able to digest the RNA (leftmost columns), indicating that the procedure isolates membrane-protected RNA, a general property of EVs.

To determine whether the RNA isolated using the spin columns was sequestered in EVs, we treated the column-bound vesicles with either RNase A, the detergent Triton X-100 (to disrupt the vesicles’ lipid bilayer), both RNase A and Triton X-100 or buffer alone (mock treatment), and measured the levels of various RNAs by RT-qPCR (Fig 4). When treated with RNase A, but not Triton, the PCR signal for the tested RNAs remained within the margin of error compared to the signal for vesicles mock treated with buffer. This suggests that RNAs captured by the column were located inside vesicles, and therefore protected by the lipid bilayer from RNase A digestion. Conversely, treating with Triton and RNase A led to near-total digestion of the RNAs, confirming that their initial resistance to digestion was due to sequestration within vesicles. Treating the membrane with Triton alone resulted in some degradation of RNA, supposedly because disruption of the vesicle membrane exposes the RNA to residual RNase activity present on the membrane, but not nearly to the same extent as when both Triton and additional RNase were applied.

### EV RNA contains high-quality total RNA including full length mRNAs and ncRNAs

**Fig 5:**
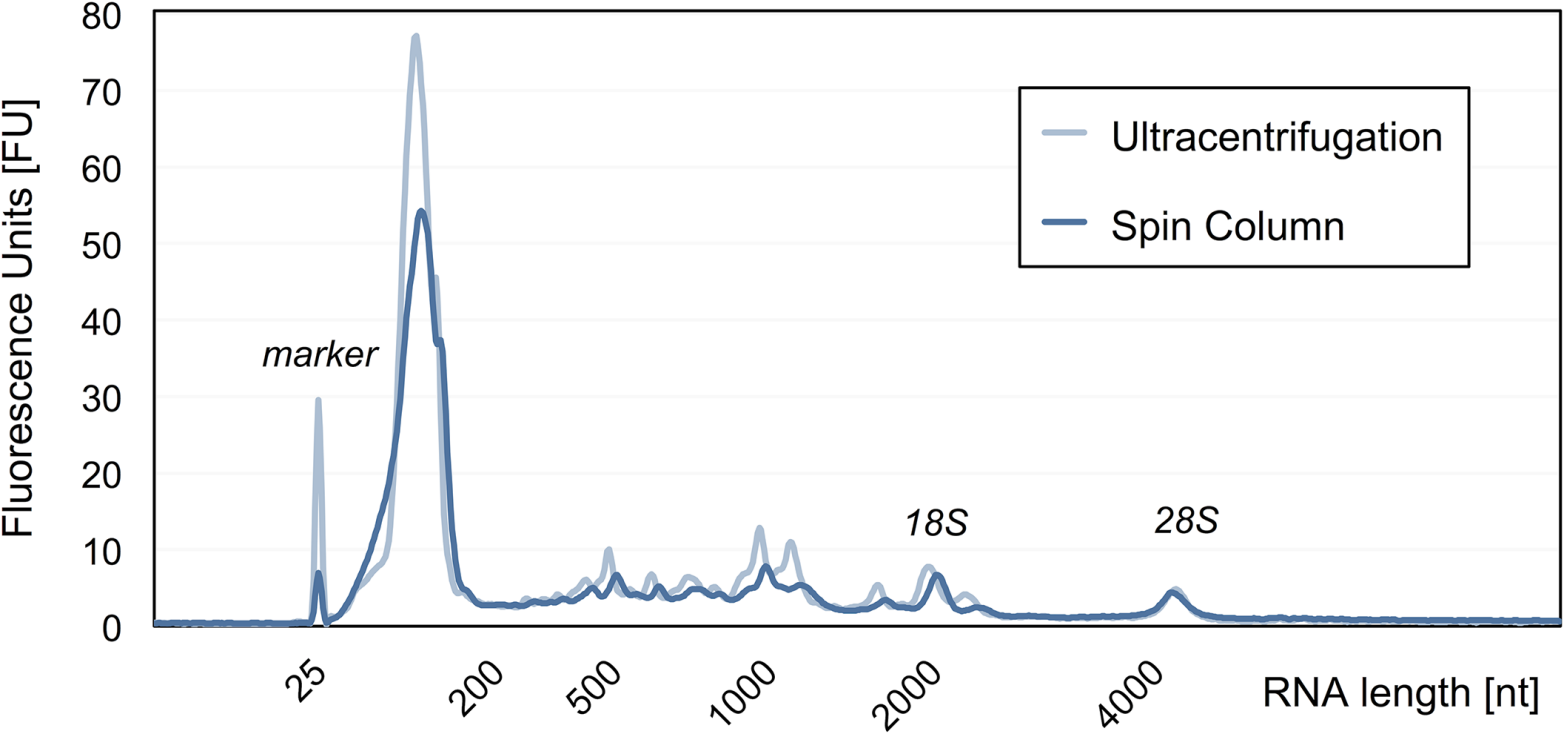
Size distribution of total RNA from cancer patient plasma isolated by membrane affinity columns and ultracentrifugation. Bioanalyzer sizing of vesicle-derived RNA purified by two methods. Total EV RNA from 2 mL plasma of a melanoma patient was isolated using membrane affinity columns and compared total EV RNA from ultracentrifugation, the current gold standard of EV isolation. Both methods purify RNA of similar size and yield.

Obtaining high-quality RNA is a critical step in performing many molecular techniques such as RT-qPCR and transcriptome analysis by next-generation sequencing or hybridization to microarrays. For biomarker discovery and diagnostic development, the quality and reproducibility of the RNA sample preparation, especially for clinical samples, is paramount. To determine the RNA quality achieved with the spin column method, we compared the Bioanalyzer profile of RNA isolated by the new method to that of RNA obtained from an optimized ultracentrifugation protocol for EV isolation. Typically, both methods yielded RNA of equivalent size distribution and abundance, as judged by analysis of a Bioanalyzer RNA Pico assay (Fig 5). The RNA yield of the spin column method generally is 1–10 ng/mL plasma, as also previously reported for ultracentrifugation [4], but varying greatly from sample to sample. An example cohort of 57 plasma extractions resulted in a median value of 7 ng/mL plasma with individual samples ranging from 1.6 to 18.2 ng/mL plasma. In the Bioanalyzer trace profile of the total RNA preparation, the majority of transcripts appeared around an approximate size of 125 bp, but many transcripts of larger sizes are clearly visible. Although the plasma sample had been pre-filtered with a 0.8 μm microfilter to remove any contaminating cellular debris, the Bioanalyzer profiles showed that both methods yield ribosomal RNA (rRNA), visible as peaks around 2 kb for the 18S and around 5 kb for the 28S subunit. These peaks become much more apparent in the electropherogram, when analyzing the small and large fraction of the same total RNA preparation as separate samples (S1 Fig), indicating that the RNA preparation indeed contains intact, full-length transcripts.

**Fig 6:**
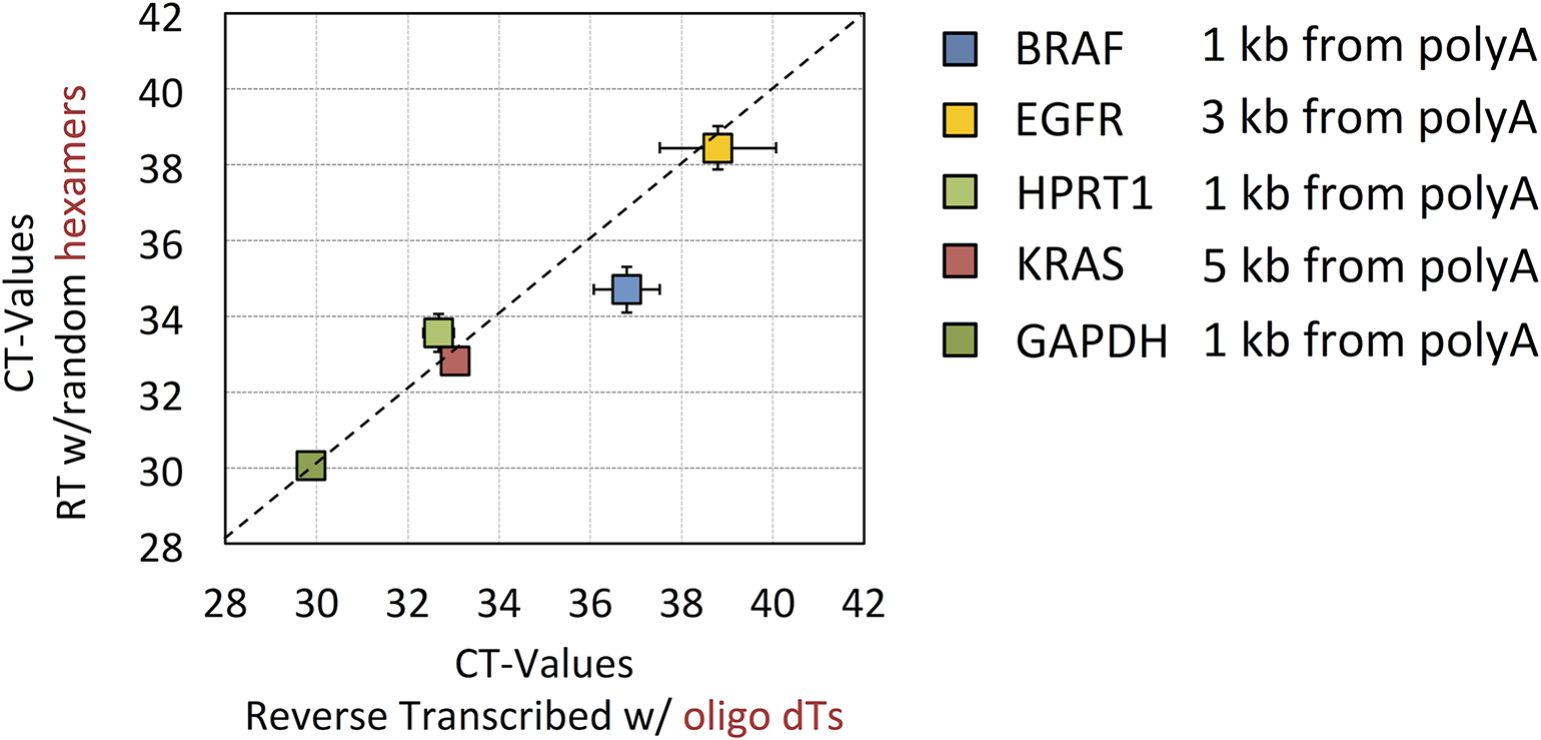
Reverse transcription of isolated total RNA using random primers and oligo-dT. RNA from 2 mL of pre-filtered plasma was extracted with membrane affinity columns and reverse transcribed by superscript II using either oligo-dT 20mers, for priming of transcripts with intact poly-A tails, or random hexamers, for priming of both degraded and non-degraded transcripts. The scatterplot shows mean and SD of 6 independent RT reactions measured by 2 qPCR replicates for each of the two datasets. The distance of each RT-qPCR assay to the poly-A tail of the transcript are noted next to the plot. A good correlation of the raw C_T_ values from random and oligo-dT priming demonstrates that the vast majority of assayed EV transcripts are intact and full-length.

In a further effort to investigate whether the RNA extracted with the method was degraded or full length we subjected the RNA to two different protocols for reverse transcription–random priming and oligo-dT priming (Fig 6). The random priming approach reverse transcribes all areas of the mRNA transcripts regardless of whether the transcripts are intact or fragmented. Oligo-dT priming on the other hand has a bias towards initiating reverse transcription from the 3’-poly-A tail of mRNAs and will only reverse transcribe the most 5’-regions if the transcripts are intact and allow the reverse transcription to run all the way. Using qPCR assays targeted against transcript regions far towards the 5’-end (Fig 6, legend) we observed no significant difference in CT value between the two reverse transcription methods, indicating that the mRNAs from the RNA preparation are largely intact, full length transcripts.

### EV RNA contains all plasma mRNAs and a specific population of miRNAs

**Fig 7:**
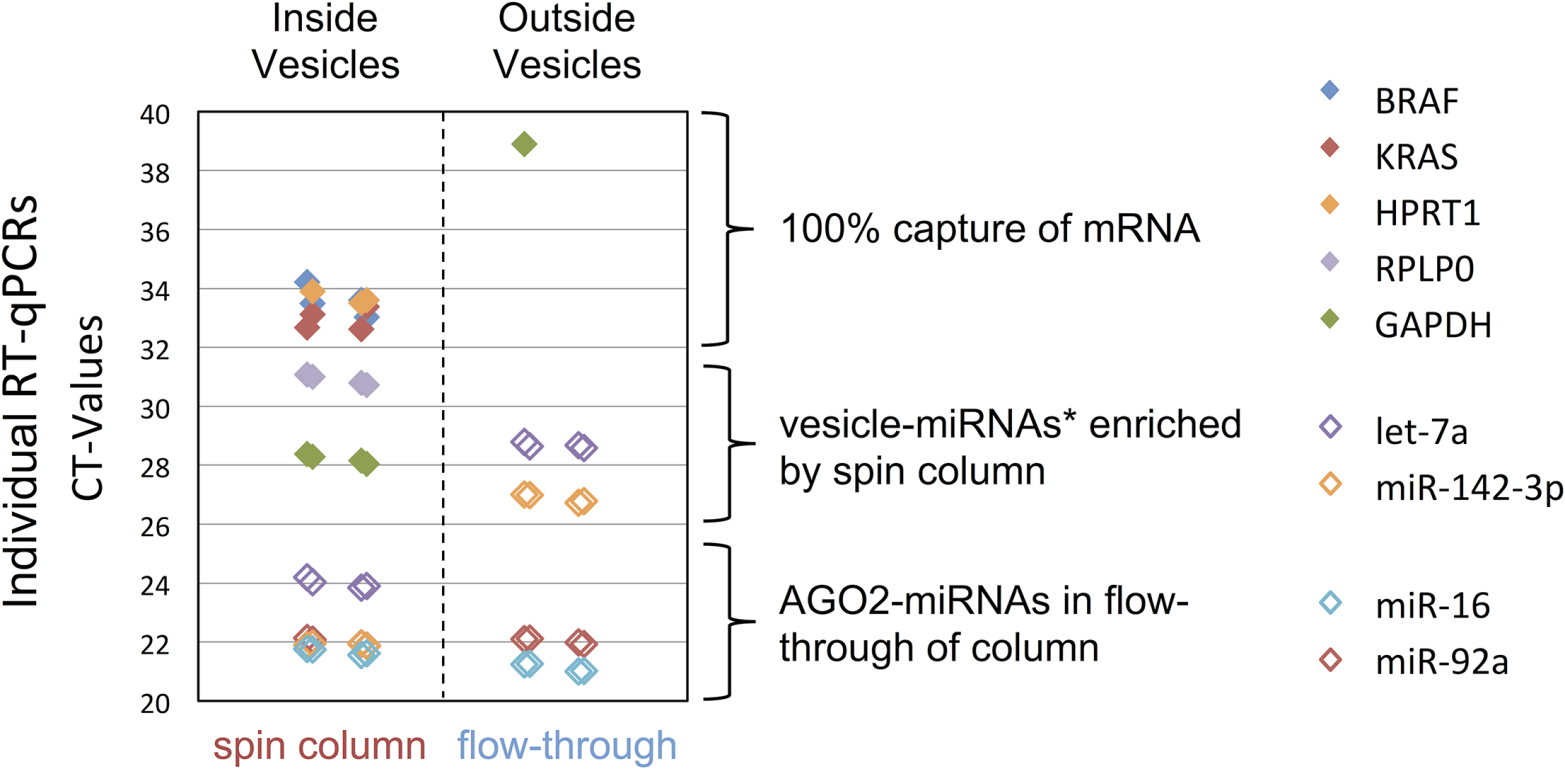
Recovery of mRNAs and miRNAs from plasma. EV RNA from 0.2 mL of pre-filtered plasma was isolated using a membrane affinity column and RNA from the flow-through of the spin column was extracted using direct lysis with the miRNeasy Serum/Plasma kit. Shown are raw C_T_ values from RT-qPCRs with rows as replicate isolations and colored diamonds as replicate qPCRs. Comparing the two fractions shows that the membrane affinity columns capture almost all mRNA and vesicle-specific miRNAs in plasma.

To characterize which types of RNA are isolated using membrane affinity columns, we used the minimum sample input amount, 0.2 mL, and performed RT-qPCR on 10% of the recovered cDNA (Fig 7, “spin column”). We achieved robust raw C_T_ values for both small and large RNAs, including RNAs important for cancer research such as BRAF and KRAS, as well as specific miRNAs like let-7a and miR-142-3p that have previously been reported to be specifically enriched in EVs [22]. Moreover, the flow-through from a 200μL aliquot of plasma after running it over the membrane affinity column was completely depleted of mRNA as judged by the RT-qPCR signal from RNA isolated after direct lysis (Fig 7, “flow-through”). The majority of signal detected in the flow-through consists of miRNAs known to be Ago2-bound and “free-circulating” outside of EVs *i*.*e*. miR-16 and miR-92a [22]. Together, these results indicate that all plasma mRNAs and specific miRNAs that are contained in vesicles are captured by the spin column method.

### Column-based purification is suitable for routine extraction of high sample volumes

**Fig 8:**
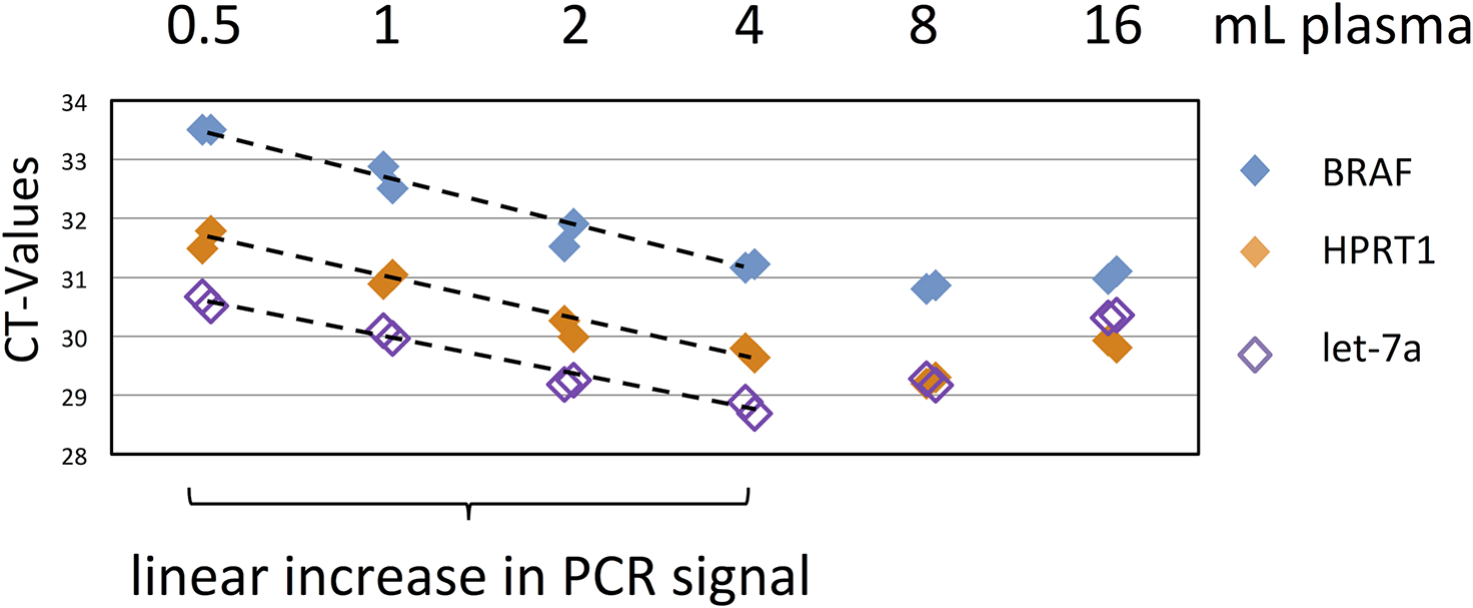
Quantification of RNA yield with increasing plasma volumes. EV RNA from increasing plasma volumes was extracted with membrane affinity columns and analyzed using RT-qPCR against two mRNAs (BRAF, HPRT1) and a miRNA known to be present in vesicles (let-7a). Shown are raw C_T_ values with rows as individual extractions and colored diamonds as replicate qPCRs. The signal increase of 1 CT with each doubling of input amount into extraction demonstrates a linear efficiency of EV extraction up to volumes of 4 mL plasma.

The spin column method was developed to be scalable from small volumes of 200 μL or less (when sample volumes are limited or the transcript of interest is believed to be present at a high copy number), up to larger volumes of 4 mL or more (for array- or sequencing-based biomarker discovery or for rare transcript detection). To this end, Fig 8 shows a linear increase in signal of three different RT-qPCR assays based on input from 0.5 mL to 4 mL of plasma, at which point the spin column membrane becomes saturated and the RNA yield does not increase further. Thus, this method can successfully handle sample volumes up to 4 mL without performance loss. Another important feature for routine use is the stability of the RNA during storage. While RNA isolations from tissue or CTCs require stabilization reagents to prevent degradation, RNA enclosed in vesicles is remarkably stable without any preservation at all.

**Fig 9:**
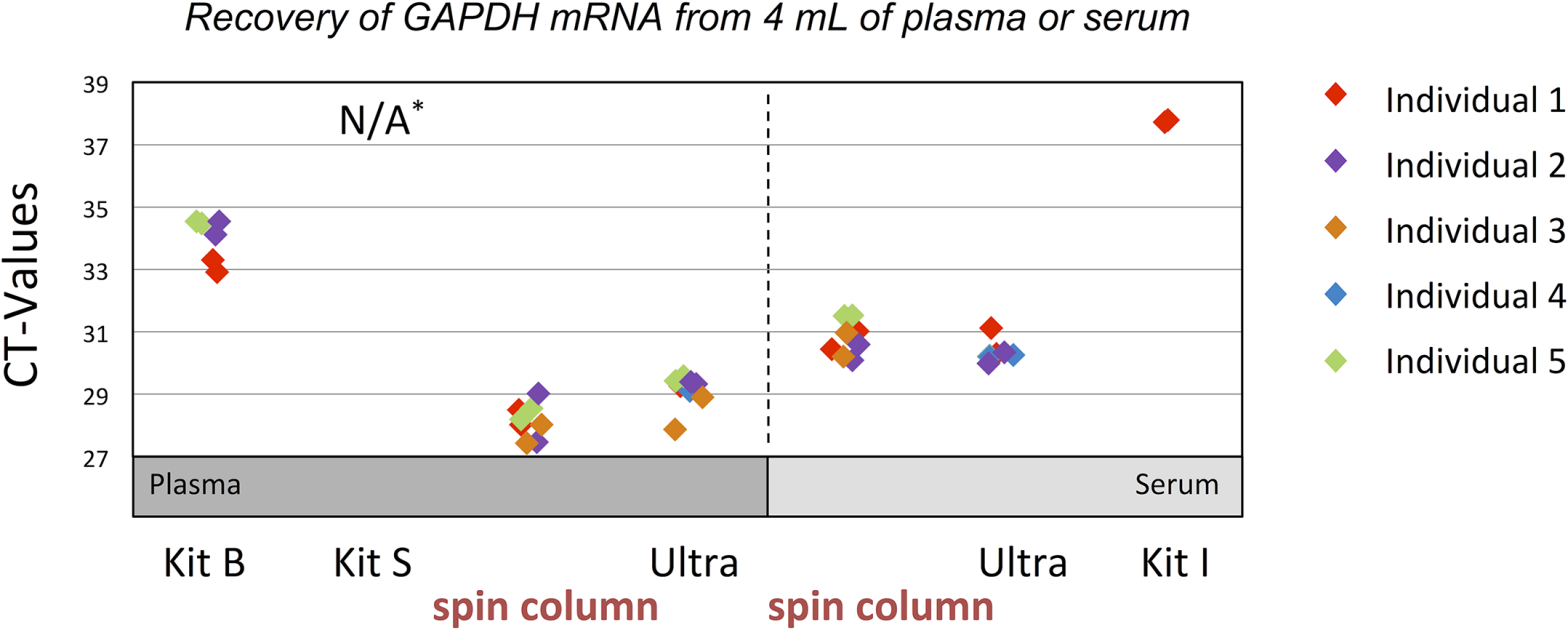
RNA extraction from high volumes of plasma and serum using several available commercial kits. EV RNA from 4 mL of plasma or serum was isolated using membrane affinity (spin column), ultracentrifugation (Ultra) and three commercially available methods based on filtration (Kit B) or polymer-based precipitation (Kit S, Kit I) according to the manufacturers recommendations. The method marked with a star had no procedure for processing of high volumes available. The plot depicts raw C_T_ values of 5 different individuals using an RT-qPCR assay against the GAPDH mRNA. Only column-based purification and ultracentrifugation efficiently recover RNA from high sample volumes.

Several EV isolation kits using a variety of different methods are currently available; we compared the methods of the different kits for yield and detection of specific genes by RT-qPCR, including the widely used GAPDH gene (Fig 9). For each kit, 4 mL of plasma or serum of five individual donors was used; some kits are specified to only be used on plasma; other kits only on serum. The samples were isolated and extracted according to the protocol provided by the kit manufacturer, and the output compared with EV isolation by ultracentrifugation and the spin column method. Both, ultracentrifugation and the spin column method, recover comparable C_T_ values for *GAPDH* in all individuals, again pointing to the complete recovery of mRNA by membrane affinity capture in serum and plasma (Fig 9). The other methods tested, based on precipitation or filtration, were not able to achieve similar RNA yield from a 4 mL sample volume.

### EV RNA can be used to detect expression of cancer pathway genes

**Fig 10:**
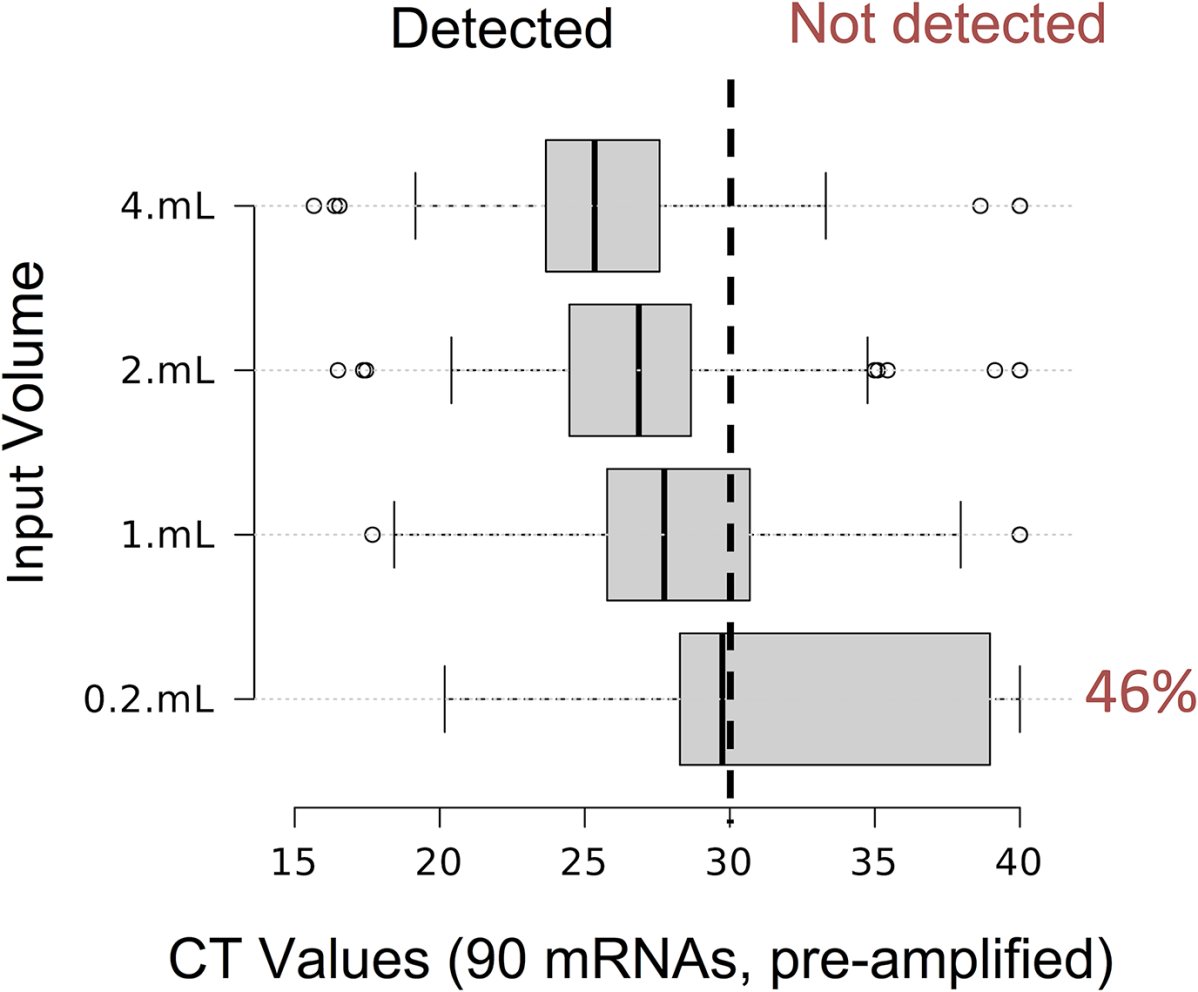
Detection of cancer-related genes in EV RNA from high volumes of plasma. EV RNA from various volumes of plasma was isolated with membrane affinity columns, reverse transcribed and pre-amplified using the RT^2^ PCR system and detected using the Human Cancer Pathway Finder PCR Array. The boxplots depict the median and interquartile range of the obtained CT values. At the lowest input volume of 0.2 mL only 46% of the mRNAs are robustly detected (CT<30) but the isolation of higher volumes leads to a linear increase in CT signal and a corresponding rate of mRNA detection. Most of the assayed oncogenes are readily detected in plasma volumes equal or higher 2mL.

Detecting mRNAs from oncogenes is a key goal of EV-associated RNA isolation, for the development of non-invasive diagnostic biomarkers. We tested for the presence of potentially interesting cancer pathway genes in 0.2 to 4 mL of plasma using the *RT* ^*2*^ *PCR PreAMP system* and *RT* ^*2*^ *Profiler Human Cancer Pathway Finder PCR Arra*y. When using enough plasma volume as input for the extraction, we were able to detect virtually all cancer genes present on the array (Fig 10). This demonstrates the feasibility of mRNA-based biomarker analysis using the spin column method (see also S1 Table). Moreover, the PCR signal linearly scales with sample input (also compare Fig 8) resulting in a much more robust detection of cancer-associated mRNAs in high volume samples. In contrast, 200 μL of sample volume is barely sufficient to detect even half of the genes assayed on the array. This emphasizes the importance of the method’s ability to process large sample volumes when conducting research on samples with low absolute amounts of the RNA molecules of interest (e.g. many cancer related genes).

## Conclusions

EV-associated RNAs are emerging as key molecules in biomarker development, and a standardized quick, reliable method to isolate such RNAs will be important in harnessing their potential. We have characterized the performance of newly developed membrane affinity spin columns to isolate EVs and extract their RNA contents from plasma and serum in an easy and reproducible workflow, which is suitable for routine clinical laboratory use with high sample volumes on many parallel extractions (Figs 8 and 9). The method purifies EVs from biofluids by membrane affinity to bind all extracellular vesicles, including exosomes. We found that the spin column is able to recover intact, regular shaped vesicles (see Figs 2 and 4) that make up only a minor fraction of all the particles present in biofluids (Table 1). The column flow-through seems to mainly consist of smaller particles, presumably lipoprotein or protein complexes (Table 1), which make up the majority of plasma particles and do not contain detectable mRNAs (Fig 5). The column membrane captures a highly purified fraction containing the EVs and enables RNA extraction of high quality (see Figs 5 and 6) that is difficult to achieve using alternative methods of isolation (Fig 8). Efficient EV purification before lysis and extraction is paramount to RNA quality, since plasma and serum contain vast amounts of RNases, potentially even elevated in clinical samples from cancer patients [48]. Before sample extraction, however, the RNA cargo is efficiently protected by the vesicle membrane (Fig 4). Therefore, while RNA isolation from tissue or CTCs requires immediate preservation to prevent degradation, RNA enclosed in vesicles is remarkably stable without any preservation at all–even during extreme storage times or unduly harsh treatment of the sample material (S2 Fig, [2]). This tremendous stability of vesicles, especially in biofluids like serum and plasma, is clearly one of the most powerful features of EV-based diagnostics. An efficient use of sample material is of interest to all research that is based on limited and finite resources like clinical samples. The spin column method virtually depletes small volumes of plasma of all mRNAs (Fig 7), with an RNA yield that scales linearly with input volumes up to 4 mL (Fig 8, Fig 10) and performs equal or better than EV isolation by ultracentrifugation (Fig 9). The aforementioned high concentrations of RNases in plasma make it plausible that indeed all mRNA in circulation is found in EVs, making EVs the main source of mRNA available for analysis of cancer-related gene expression from blood (Fig 10). A specific and abundant population of miRNAs, however, can be found outside of vesicles, considered to be “free circulating”, bound to and protected from RNases by Ago2-proteins [22]. Since the membrane affinity column can be used to deplete plasma of miRNAs located inside vesicles (Fig 7), the flow-through of the spin column might be of particular interest to research on Ago2-bound miRNAs, thought to be released into the blood stream during apoptosis and necrosis [37]. In contrast, currently available methods based on precipitation and ultracentrifugation can be prone to contamination with Ago2-bound miRNAs due to co-pelleting and co-precipitation during the isolation process [45].

In summary, our data show that EV purification by membrane affinity spin columns exhibits several characteristics that markedly improve on current methods for isolating RNA from EVs. The new method demonstrates equal or better yields and purity of RNA compared to existing products and protocols while accommodating a wide range of sample volumes, enabling detection of low-abundance transcripts. It specifically isolates RNA contained within EVs while excluding protein-associated circulating RNA and provides an easy, fast workflow that is suitable for routine laboratory use.

## Supporting Information

S1 Fig Size fractionation of total RNA from extracellular vesicles. Total EV RNA from 2 mL of pre-filtered plasma was isolated by membrane affinity columns, bound to an RNeasy column using 70% ethanol, eluted with water and analyzed using a Bioanalyzer RNA Pico assay (1). The same RNA sample was fractionated by binding the large RNAs to a second RNeasy column using 350 μL RLT and ethanol up to a final concentration of 20% (2) The small RNAs present in the flow-through were isolated using a third RNeasy column with a final concentration of 70% ethanol in the binding step (3). The presence of sharp ribosomal RNA peaks in the Bioanalyzer profile (*) demonstrates the purification of large, intact, non-degraded RNAs from EVs.
(TIFF)

S2 Fig Robust detection of EV RNA following prolonged storage and multiple freeze/thaw cycles. (**A**) EV RNA from 1.5 mL of pre-filtered plasma of a patient with ovarian cancer, stored at -80°C for 12 years, was isolated using ultracentrifugation and analyzed with a Bioanalyzer RNA Pico assay. The presence of sharp ribosomal RNA peaks in the Bioanalyzer profile (*) demonstrates the purification of large, intact, non-degraded RNAs from EVs. (**B**) To assess RNA degradation caused during repetitive freeze-thaw cycles, plasma samples were subjected to up to nine cycles of thawing and refreezing to -80°C, and 2 mL aliquots were analyzed using membrane affinity columns and RT-qPCR. Shown are raw CT values with rows as individual extractions and colored diamonds as replicate qPCRs. No significant change in CT value can be detected, pointing to a certain stability of EVs during freeze/thaw cycles. (**C**) To assess RNA degradation during prolonged storage at room temperature, samples were allowed to sit on the bench at room temperature (25°C) for between 0 and 42 hours and 2 mL aliquots were analyzed using membrane affinity columns and RT-qPCR. There was virtually no difference in the detection of the genes over the course of a 2-day incubation.
(TIFF)

S1 Table RT-qPCR CT Values from Human Cancer Pathway Finder PCR Array performed on increasing amounts of plasma EV RNA. (XLSX)
