# Supplementary figures and images for: Characterization of RNA from Exosomes and Other Extracellular Vesicles Isolated by a Novel Spin Column-Based Method
Source: PLoS One. 2015 Aug 28;10(8):e0136133. doi: 10.1371/journal.pone.0136133 (PMC4552735; doi:10.1371/journal.pone.0136133)

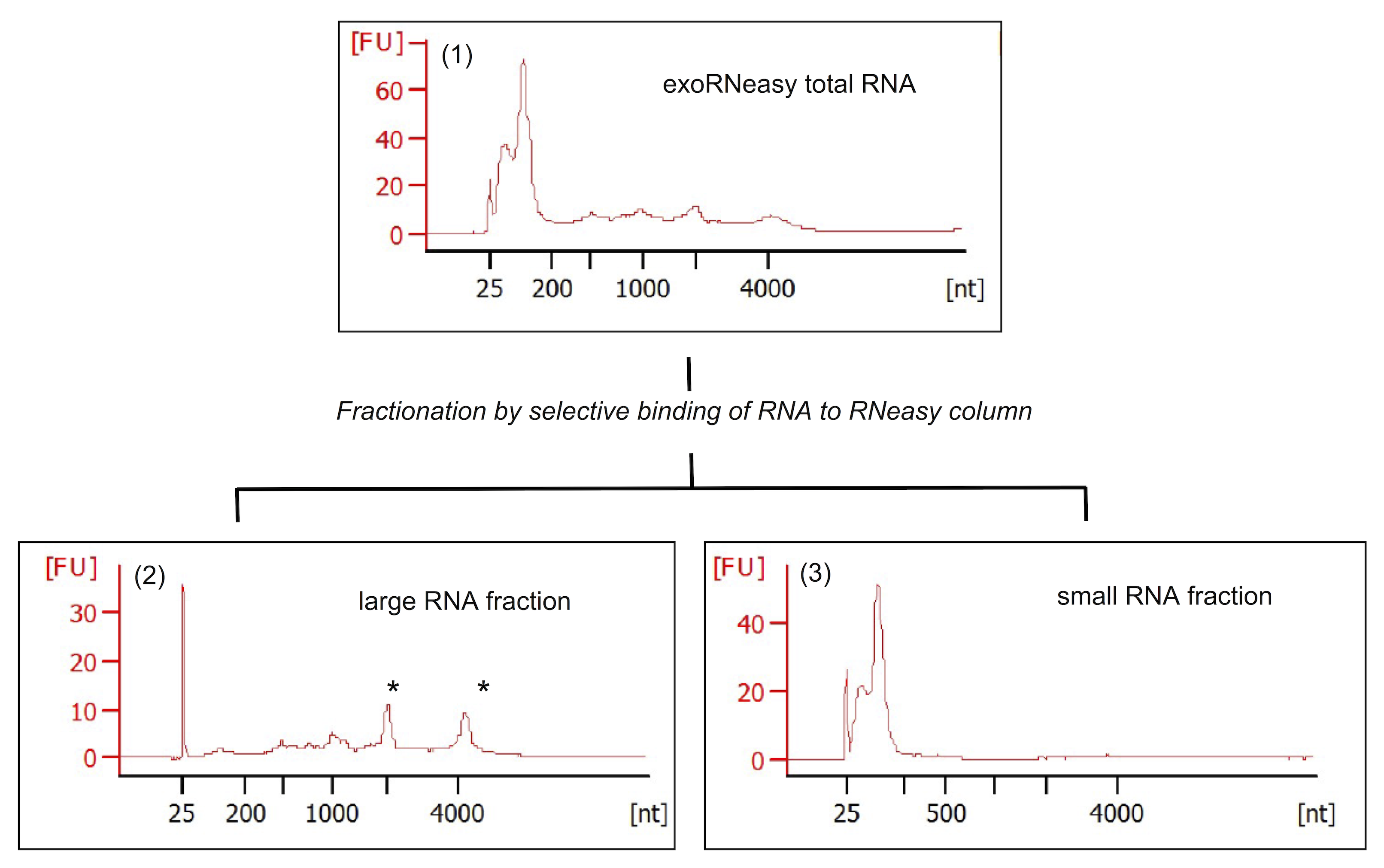

Supplement: S1 Fig — Total EV RNA from 2 mL of pre-filtered plasma was isolated by membrane affinity columns, bound to an RNeasy column using 70% ethanol, eluted with water and analyzed using a Bioanalyzer RNA Pico assay (1). The same RNA sample was fractionated by binding the large RNAs to a second RNeasy column using 350 μL RLT and ethanol up to a final concentration of 20% (2) The small RNAs present in the flow-through were isolated using a third RNeasy column with a final concentration of 70% ethanol in the binding step (3). The presence of sharp ribosomal RNA peaks in the Bioanalyzer profile (*) demonstrates the purification of large, intact, non-degraded RNAs from EVs. (TIFF) [file pone.0136133.s001.tiff]

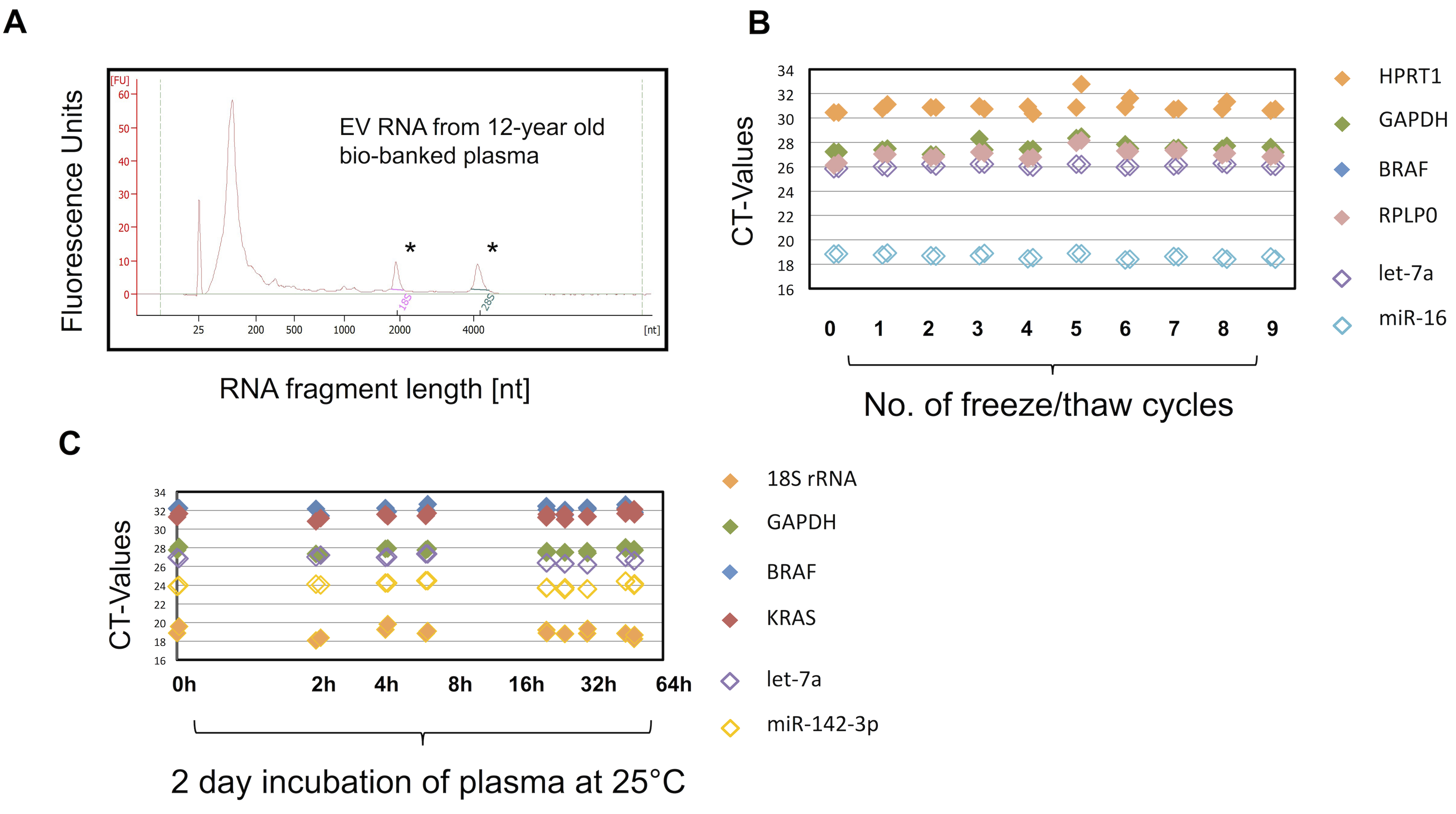

Supplement: S2 Fig — (A) EV RNA from 1.5 mL of pre-filtered plasma of a patient with ovarian cancer, stored at -80°C for 12 years, was isolated using ultracentrifugation and analyzed with a Bioanalyzer RNA Pico assay. The presence of sharp ribosomal RNA peaks in the Bioanalyzer profile (*) demonstrates the purification of large, intact, non-degraded RNAs from EVs. (B) To assess RNA degradation caused during repetitive freeze-thaw cycles, plasma samples were subjected to up to nine cycles of thawing and refreezing to -80°C, and 2 mL aliquots were analyzed using membrane affinity columns and RT-qPCR. Shown are raw CT values with rows as individual extractions and colored diamonds as replicate qPCRs. No significant change in CT value can be detected, pointing to a certain stability of EVs during freeze/thaw cycles. (C) To assess RNA degradation during prolonged storage at room temperature, samples were allowed to sit on the bench at room temperature (25°C) for between 0 and 42 hours and 2 mL aliquots were analyzed using membrane affinity columns and RT-qPCR. There was virtually no difference in the detection of the genes over the course of a 2-day incubation. (TIFF) [file pone.0136133.s002.tiff]
